# Radiomics, deep learning and early diagnosis in oncology

**DOI:** 10.1042/ETLS20210218

**Published:** 2021-12-07

**Authors:** Peng Wei

**Affiliations:** Department of Biostatistics, The University of Texas MD Anderson Cancer Center, Houston, Texas, U.S.A.

**Keywords:** cancer, deep learning, early detection, medical imaging, radiomics

## Abstract

Medical imaging, including X-ray, computed tomography (CT), and magnetic resonance imaging (MRI), plays a critical role in early detection, diagnosis, and treatment response prediction of cancer. To ease radiologists’ task and help with challenging cases, computer-aided diagnosis has been developing rapidly in the past decade, pioneered by radiomics early on, and more recently, driven by deep learning. In this mini-review, I use breast cancer as an example and review how medical imaging and its quantitative modeling, including radiomics and deep learning, have improved the early detection and treatment response prediction of breast cancer. I also outline what radiomics and deep learning share in common and how they differ in terms of modeling procedure, sample size requirement, and computational implementation. Finally, I discuss the challenges and efforts entailed to integrate deep learning models and software in clinical practice.

## Introduction

A variety of medical imaging modalities, including ultrasound, X-ray, computed tomography (CT), and magnetic resonance imaging (MRI), have played a critical role in early detection, diagnosis, and treatment response prediction of cancer. For example, it is recommended that average-risk women of age 45 years or older have a screening mammography, which is an X-ray imaging of the breast, every year to screen for breast cancer [1], while heavy smokers aged 50 to 80 years are recommended to have annual screening for lung cancer with low-dose CT [2]. Early detection of cancer entails screening and early diagnosis, two related yet subtly different notions. Cancer screening is aimed to identify asymptomatic cancer or pre-cancerous lesions in a target population without symptoms, and early diagnosis is aimed at identifying symptomatic cancer cases at the earliest possible stage [3]. For example, a woman who undergoes annual screening mammography with a suspicious lump will be followed up with a diagnostic mammography and further a biopsy for pathological confirmation. The goal of both screening and early diagnosis is to identify cancer early so that the cancer is still localized and can be treated or cured, leading to improved survival and reduced mortality. In this mini-review article, I will use breast cancer as an example and review how medical imaging and its quantitative modeling, including radiomics [4,5] and deep learning [6], have improved the early detection and treatment response prediction of breast cancer.

## Medical imaging in early detection of breast cancer

**Figure 1.**
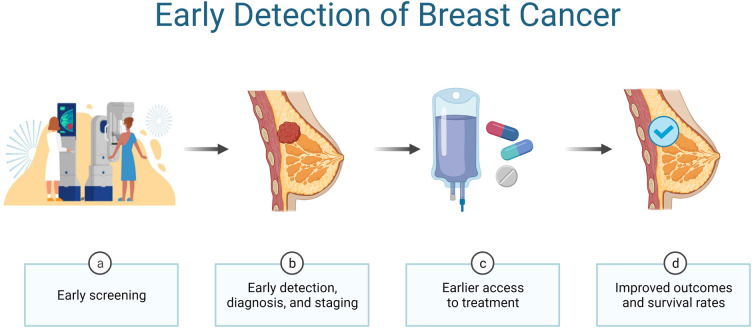
Early detection of breast cancer (created with BioRender.com).

Female breast cancer is the most commonly diagnosed cancer with an estimate of 2.3 million new cases and over 680 000 new deaths worldwide in 2020 [7]. As demonstrated in Figure 1, early detection via screening mammography remains the most effective way to reduce the mortality due to breast cancer [1]. In the US, annual screening mammography is recommended for average-risk of women aged 40 years or older [1]. As a standard clinical practice, fellowship-trained diagnostic radiologists read and interpret medical imaging to make qualitative or semi-quantitative ascertainment of the presence of malignancy. For instance, radiologists in the US classify each screening mammography into several ordinal categories, ranging from negative (category 1), benign (category 2) to suspicious (category 4) and highly suggestive of malignancy (category 5) according to the Breast Imaging Reporting and Data System (BI-RADS) developed by the American College of Radiology (ACR) [8]. While the current screening mammography has achieved 86.9% sensitivity and 88.9% specificity as reported recently [9], there is still room for performance improvement. In particular, for women with extremely dense breasts (∼10% of women), i.e. those with much higher proportion of fibroglandular (dense) tissue compared with fatty (nondense) tissue in the breast, both fibroglandular tissue and tumor look white on a mammography, making it difficult to discern a small tumor and leading to poor diagnostic accuracy. To ease radiologists’ task and help with challenging cases, computer-aided diagnosis (CAD) has been developing rapidly in the past decade, pioneered by radiomics early on, and more recently, driven by deep learning. I will review radiomics, deep learning, and their applications in medical imaging in the next sections.

## Radiomics and its applications in medical imaging

**Figure 2.**
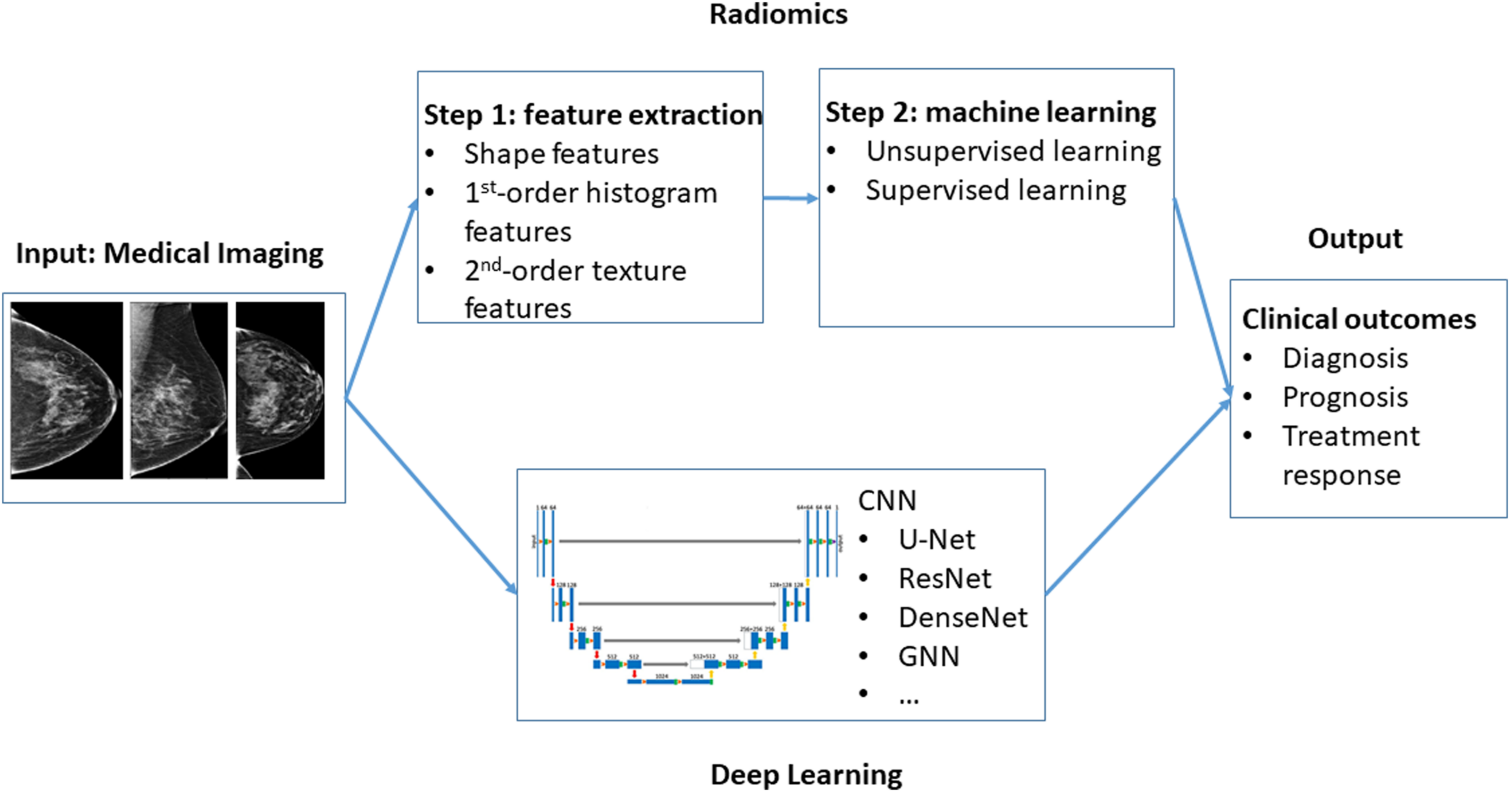
Comparison of radiomics and deep learning in quantitative modeling of medical imaging. CNN: convolutional neural network; GNN: graph neural network.

Radiomics in medical imaging refers to the extraction of a large number (typically in the hundreds) of quantitative features, such as shape features (e.g. volume and maximum diameter), first-order histogram features (e.g. mean, median, minimum, maximum, skewness, and kurtosis of intensity distribution), and second-order texture features that capture spatial arrangement of the voxel intensities [6]. The feature extraction process is essentially a series of linear and non-linear dimension reduction in the raw medical imaging [10,11]. As illustrated in Figure 2, The resulting quantitative features are then subject to statistical and machine learning modeling, such as unsupervised learning (e.g. hierarchical clustering) to discover tumor subtypes, and supervised learning (e.g. penalized regressions [12] and boosting [13]) to predict presence of tumor (diagnosis), patient survival (prognosis), or treatment response [5]. For example, mammographic texture features, a group of 29 well annotated radiomic features [14], have be shown to be strong independent risk factors for breast cancer and further improve risk prediction when combined with breast density [15].

Radiomics analysis of MRI imaging has also been used to predict treatment response in triple negative breast cancer (TNBC). TNBC is a subtype of breast cancer that accounts for ∼20% of all breast cancers and is characterized by lacking estrogen receptor, progesterone receptor, and HER2 expression [16]. Compared with other breast cancer subtypes, TNBC is more aggressive and is associated with a higher rate of relapse and a lower rate of overall survival. Patients with TNBC usually undergo neoadjuvant systemic therapy (NAST) for downstaging of disease to facilitate less invasive surgery. The extent of downstaging is used as a surrogate prognostic marker [17]. Pathological complete response (pCR) to NAST is only seen in about half of the TNBC patients undergoing the treatment. To this end, ‘A Robust TNBC Evaluation FraMework to Improve Survival’ (ARTEMIS) is an ongoing clinical trial (ClinicalTrials.gov identifier: NCT02276443) aimed at using longitudinal genomic and imaging signatures to identify TNBC patients who are sensitive/insensitive to standard NAST chemotherapy. TNBC patients of stage I-III in the study undergo biopsy before treatment, and then immediately begin NAST. As part of the clinical trial protocol, multiple sequences of MRI imaging, such as T1, T2, ultrafast Dynamic Contrast Enhanced (DCE)-MRI [18], and Chemical Exchange Saturation Transfer (CEST)-MRI [19], are performed at baseline, after two cycles, and at completion of four cycles of NAST. pCR is then ascertained by pathologists following appropriate surgery according to the size of the residual tumor. To investigate if radiomic phenotypes can predict pCR, 390 radiomic features (first-order histogram features and second order grey-level-co-occurrence matrix) were extracted from each of 74 patients with Stage I-III TNBC who underwent ultrafast DCE-MRI at baseline in the ARTEMIS trial [20]. Given the relatively small sample size and large number of radiomic features, 3-fold cross-validation and a penalized logistic regression model with the elastic net penalty were used to build prediction models. The elastic net penalty is appealing in that it enables feature selection while accommodating potentially correlated features as is the case for radiomics [12]. The resulting radiomic model consisting of 24 features had an area under the receiver operating characteristics curve (AUC) of 0.80 for predicting pCR. This model is being integrated with longitudinal MRI at two cycles and four cycles of NAST, and further validated in an independent set of ARTEMIS patients.

## Deep learning and its applications in medical imaging

Deep learning is a family of machine learning and artificial intelligence methods based on multilayered artificial neural networks (ANNs) [21] and has been shown to outperform classical supervised learning methods in many applications such as face recognition [22], chess (AlphaGo), protein 3D structure prediction [23], and intelligent medical diagnostics [24,25]. As the building block of deep learning, an ANN is a non-linear mathematical model that allows a complex relationship between the input data and the output [21]. A deep neural network is an ANN with many hidden layers, commonly 10+ and even 100+ layers, while a convolutional neural network (CNN) is a specialized type of deep neural network designed for image data. A CNN performs a linear operation called a ‘convolution’ that is capable of capturing the spatial dependency in an image. Commonly used CNN models include U-Net [26], ResNet [27], DenseNet [28], and Graph CNN [29]. Deep learning has been successfully applied to screening mammography with superior performance. Yala et al. [30] applied the ResNet to 88, 994 full-field screening mammograms and developed 5-year breast cancer risk prediction models: the mammography-based deep learning risk model had an AUC of 0.68, compared with an AUC of 0.62 by a breast density-only risk model. McKinney et al. [25] developed a deep learning model for identifying breast cancer in screening mammograms, which had an AUC greater than that for the average of six radiologists by an absolute margin of 11.5%. More recently, Lotter et al. [6] applied the ResNet to over 100 000 2D and 3D screening mammograms from 5 sites in the US and China and the resulting deep learning model had AUCs ranging from 0.922 to 0.971 and outperformed five out of five radiologists, achieving a 14.2% greater sensitivity and a 24% increase in specificity. On the other hand, Shu et al. [31] approached computational modeling of screening mammography from a different angle and developed a deep learning approach to re-create rarely stored for-processing (raw) digital mammograms from routinely stored for-presentation (processed) mammograms. ‘Raw’ images generated by full-field digital mammography are digitally manipulated to enhance some features, such as contrast and resolution, to produce ‘for-presentation’ images that are optimized for visual cancer detection by radiologists. Raw images are more appropriate for quantitative analysis, such as breast density and texture analysis, than processed images. However, in clinical settings, raw images are rarely archived due to cost and storage constraints. Moreover, mammography equipment manufacturers do not disclose their raw-to-processed-image conversion steps, and inversion algorithms are not available. To this end, Shu et al. [31] developed a deep-learning approach, based on the U-Net CNN, to re-create raw digital mammograms from for-presentation mammograms. The authors used 3713 pairs of raw and processed mammograms collected from nearly 900 women in the ‘Mammography, Early Detection Biomarkers, Risk Assessment, and Imaging Technologies’ (MERIT) cohort study (ClinicalTrials.gov Identifier: NCT03408353) at the University of Texas MD Anderson Cancer Center. The deep-learning approach performed well in recreating raw mammograms with strong agreement between the true and re-created raw images in the test dataset as measured by four image evaluation metrics, breast density calculation, and the majority of 29 widely used texture features (12 gray-level histogram features, eight co-occurrence features, seven run-length features, and two structural features) [14].

## Radiomics vs deep learning

Although both radiomics and deep learning are commonly applied in quantitative modeling of medical imaging data, they have some key differences as demonstrated in Figure 2. First, radiomics analysis can be considered as a two-step process: dimension reduction by extraction of hundreds of quantitative features capturing the tumor morphology and spatial heterogeneity in the image, followed by unsupervised learning of the radiomic features or supervised learning by correlating the radiomic features with clinical outcomes, such as cancer diagnosis, prognosis, or treatment response. On the other hand, deep learning can be considered as a one-step end-to-end supervised learning process that uses the entire image as the input and clinical outcomes as the output and allows highly flexible, non-linear, and richly parameterized relationship between the input and output as afforded by the multi-layer, inter-connected ANN. Second, as a result of the above difference, radiomics is applicable and feasible for moderate sample sizes, such as those in the hundreds, while deep learning typically requires at least thousands of training samples to avoid overfitting the data and achieve stable and superior performance. To address the latter challenge, researchers have employed transfer learning [32] to tune pre-trained deep learning models from similar datasets for a new dataset of moderate sample size [33,34]. Third, while extraction of radiomic features needs specialized software packages, such as ‘RadAR’ R package [35] and those reviewed in [11], the unsupervised and supervised learning of radiomic features can be performed in any machine learning software packages in R. On the other hand, deep learning needs more specialized software, such as Python libraries Pytorch, Tensorflow, and Keras, and hardware, such as graphics processing unit (GPU) and tensor processing unit (TPU). Finally, deep learning is much more computationally expensive than traditional radiomics analysis [36]. Take, for example, the deep learning of screening mammograms [31]. While it took several hours to train the modified U-Net for 300 epochs (training cycles) using four NVIDIA Titan Xp GPUs (total memory, 48 GB) given a set of hyper-parameters, such as learning rate and mini-batch size, tuning the hyper-parameters entailed many rounds of re-training the model. In contrast, it only takes several minutes to perform the feature extraction and downstream unsupervised/supervised learning in radiomics analysis with a comparable sample size using conventional CPU computing.

## Discussion

In this mini-review article, I have primarily focused on breast cancer and reviewed how medical imaging, including screening mammography and MRI, and their quantitative modeling, including radiomics and deep learning, have advanced the early detection and treatment response prediction of breast cancer. Substantial progresses have also been made in quantitative modeling of medical imaging for other cancer sites, such as low-dose CT screening for lung cancer [37], pathology imaging for lung cancer diagnosis [38], diagnosis and treatment response prediction of liver cancer [39,40], to name a few. Despite the promising performance of deep learning models which often outperform experienced radiologists [6,25], they have been largely developed and studied in the academic and research settings, not yet implemented and integrated in the clinical setting. To achieve the latter goal, researchers, clinicians, and government regulatory agencies will need to work together to extensively test the robustness of the deep learning models and software implementation in terms of across imaging platform and across patient population generalizability, and to train clinicians to adapt to artificial intelligence's assistance in making diagnosis and clinical decisions. Finally, radiogenomics, which integrates medical imaging and genomics data to improve cancer diagnosis and prognosis models over those based on either data type alone, is another emerging direction in quantitative modeling of imaging data [5,41] and warrants further research.

## Summary

- Medical imaging, including X-ray, computed tomography (CT), and magnetic resonance imaging (MRI), plays a critical role in early detection, diagnosis, and treatment response prediction of cancer.
- Radiomics refers to the extraction of a large number of quantitative features, such as shape features, first-order histogram features, and second-order texture features that capture spatial arrangement of the voxel intensities. The resulting quantitative features are then subject to statistical and machine learning modeling, such as unsupervised learning to discover tumor subtypes, and supervised learning for diagnosis, prognosis, or treatment response prediction.
- Deep learning is a family of machine learning and artificial intelligence methods based on multilayered artificial neural networks and has been shown to outperform classical supervised learning methods in medical imaging applications.
- Implementation and integration of deep learning models and software in clinical practice require substantial joint efforts from researchers, clinicians, and government regulatory agencies.

## Abbreviations

ANN: artificial neural networks
BI-RADS: breast imaging reporting and data system
CAD: computer-aided diagnosis
CNN: convolutional neural network
CT: computed tomography
MRI: magnetic resonance imaging
